# Smart Method for Carotenoids Characterization in *Haematococcus pluvialis* Red Phase and Evaluation of Astaxanthin Thermal Stability

**DOI:** 10.3390/antiox9050422

**Published:** 2020-05-13

**Authors:** Patrizia Casella, Angela Iovine, Sanjeet Mehariya, Tiziana Marino, Dino Musmarra, Antonio Molino

**Affiliations:** 1 ENEA, Italian National Agency for New Technologies, Energy and Sustainable Economic Development, Department of Sustainability—CR Portici. P. Enrico Fermi, 1, 80055 Portici (NA), Italy; patrizia.casella@enea.it (P.C.); angela.iovine@unicampania.it (A.I.); sanjeet.mehariya@unicampania.it (S.M.); 2 Department of Engineering, University of Campania “Luigi Vanvitelli”, Real Casa dell’Annunziata, Via Roma 29, 81031 Aversa (CE), Italy; tiziana.marino@unicampania.it (T.M.); dino.musmarra@unicampania.it (D.M.)

**Keywords:** characterization, carotenoids, chromatography, microalgae, astaxanthin, temperature, isomerization

## Abstract

*Haematococcus pluvialis* microalgae is a promising source of astaxanthin, an excellent antioxidant carotenoid. *H. pluvialis*, as well as other species, could find more extensive applications as healthy food for a variegated carotenoids composition in addition to astaxanthin. Official method has not currently been used for this purpose. The objective of this work was to propose a method to characterize carotenoids in *H. pluvialis* after the comparison between spectrophotometric and liquid chromatography analysis. In addition, in order to improve the use of astaxanthin in the food industry, thermal stability was investigated. In this context, the effect of temperature at 40–80 °C, over a 16 h storage period was tested on astaxanthin produced by *H. pluvialis*. A further test was carried out at room temperature (20 °C) for seven days. A decrease in the astaxanthin concentration was observed at all tested temperatures with a decrease >50% of all-trans isomer at 80 °C after 16 h and an increase of 9-cis and 13-cis isomers. In conclusion, the obtained results showed the importance of evaluating the degradation effect of temperature on astaxanthin used as a food additive for a future greater enhancement of this bioproduct in the food field.

## 1. Introduction

**Figure 1:**
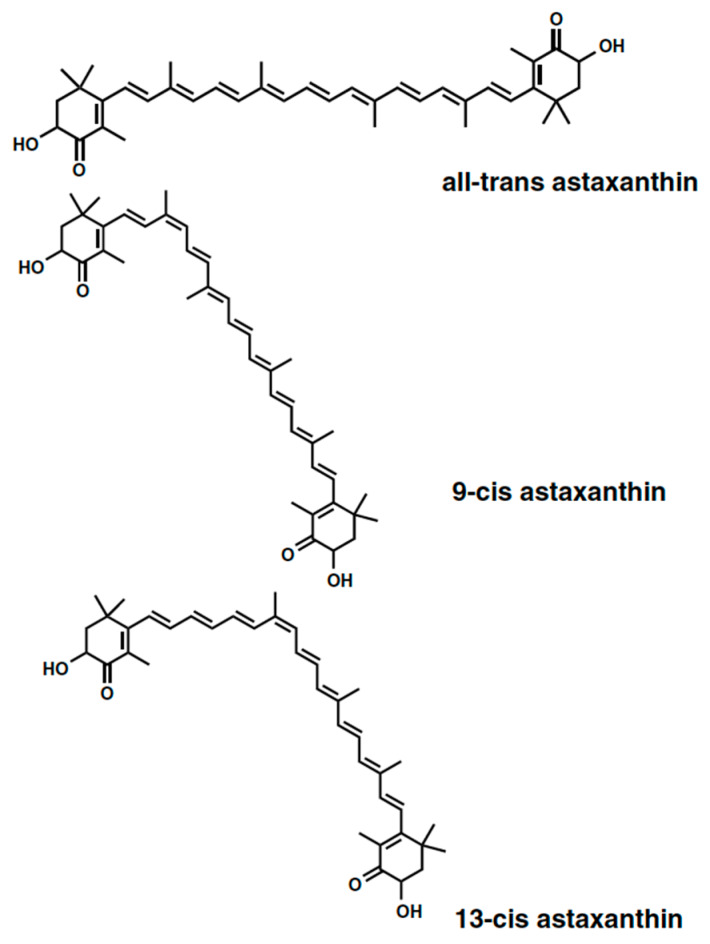
Astaxanthin isomers molecular structures. All-trans-astaxanthin; 9-cis astaxanthin; and 13-cis astaxanthin [10].

Carotenoids are a class comprised of ~600 liposoluble, thermo-labile, colored compounds that are contained in plants, fruits, and microalgae with the main function of capturing the wavelengths of light for photosynthesis and chlorophyll protection of the cell from photo damage [1,2]. In recent years, some carotenoids have found applications in food and feed markets as additives for human and animal nutrition, and as supplements in the nutraceutical industry for their natural coloring and antioxidant properties [3,4,5]. These compounds have a relatively high market value in which microalgal carotenoids have even reached a value of 1.2 billion USD with growing prospects [6]. Astaxanthin is one of the protagonists of the carotenoids market thanks to its remarkable antioxidant properties towards humans [7,8], and for its reddish-brown color that gives an attractive appearance to salmonid flesh and for the beneficial effects [9]. More precisely, astaxanthin is a xanthophyll, a carotenoid characterized by the presence of hydroxyl groups or oxygen molecules in the molecular structure, which are found in nature in the form of isomers, all-trans, 9-cis, and 13-cis (Figure 1).

Among the main sources of astaxanthin, such as *Phaffia rhodozyma* yeast and shrimps whose carapace is colored, microalgae *Haematococcus pluvialis* is one of the most attractive because astaxanthin can make up to 2%–3% dry weight. *H. pluvialis* lives in freshwater environments and during its life cycle changes from a green vegetative form (green phase), where cell division takes place, to a form of incistement (red phase), where the cell is transformed into a resistant red cyst, rich in astaxanthin [11].

Natural astaxanthin is the most powerful antioxidant among carotenoids whose antioxidant properties are also greater than beta-carotene and lutein properties. According to some studies, the antioxidant properties of astaxanthin are 11 times greater than beta-carotene, and 2.75 times greater than lutein, also exceeding the antioxidant power of vitamins A and E [12,13]. The antioxidants properties are due to the molecular structure characteristics such as polar groups and double bonds that quench free radicals and remove high energy electrons, respectively, as well as due to the functional stabilizing activity of astaxanthin in cellular membranes [14].

Antioxidant astaxanthin activity has been investigated against several high reactive molecules involved in serious cardiovascular disease. Astaxanthin has shown high protective and anti-inflammatory action against oxidative stress, thus, reducing cardiovascular disease risk [15]. Anticancer properties have also been demonstrated for astaxanthin that reduced the proliferation and the migration of cancer cells reducing the incidence of breast cancer [16]. Other health benefits have been investigated for astaxanthin such as eyes and skin protection, brain health and hypertension protection, and lipid peroxidation reduction [12].

Nowadays, astaxanthin has been recognized at the European level and by the United States Food and Drug Administration (U.S. FDA) as a coloring additive in feed for salmonids, ornamental fish and birds, and as a food supplement [17]. The U.S. FDA has authorized both the synthetic form of astaxanthin and the natural form produced by the microalgae *H. pluvialis* as a feed additive for salmonids, while the European Community has continued to authorize the synthetic form [18] and the natural form as an oleoresin produced by *H. pluvialis* as a food supplement [17]. The strength of the use of *H. pluvialis* biomass as an additive or supplement, as well as other microalgae such as *D. salina* which is rich in β-carotene, is the ability to produce more carotenoids that can constitute a mix of antioxidants and vitaminic substances and other added value compounds such as proteins, carbohydrates, lipids, and total dietary fibers as highlighted by different studies [19,20,21]. However, the simultaneous production of several carotenoids in *H. pluvialis* requires the use of appropriate analytical methods to identify these important antioxidants.

Currently, official methods for the quantification of astaxanthin, and other carotenoids in microalgae are not available. In fact, there is only an evaluation report on the spectrophotometric and chromatographic analysis of the synthetic astaxanthin additive (2a161j) [22]. This report provided a spectrophotometric analysis of astaxanthin at 431 nm in a powdery or water dispersible product and stated that the method had not been applicable to natural forms of astaxanthin derived, for example, from microalgae containing more carotenoids. In addition, the chromatographic method is applicable for the total quantification of astaxanthin, including isomers, in premixtures and feed stuffs. The methodology foresees a quantification in HPLC-UV/VIS at the normal stage, allowing the separation of astaxanthin isomers from other carotenoids that can be present.

In the literature, astaxanthin produced from *H. pluvialis* has been analyzed by spectrophotometer or by liquid chromatography. The spectrophotometric analysis was carried out with the aim of quantifying both astaxanthin and the total carotenoids. In particular, astaxanthin was detected at 480 nm using the extinction coefficient A^1%^, equal to 2500, according to the method reported by Davies (1976) [23], whereas the total carotenoids content followed the procedure reported by Lichtenthaler (1987) [24], which provided the use of specific equations to calculate the content of chlorophyll a, b, and total carotenoids depending on the extraction solvent. Both methods were applied in the literature by different authors [25,26] to quantify total carotenoids, while other authors have carried out these methods for the analysis of astaxanthin [27,28] and other carotenoids such as canthaxanthin, lutein, beta-carotene [29,30], and its isomers by HPLC-UV/VIS. A more accurate quantification of astaxanthin by spectrophotometer has been proposed by Li et al. (2012) [31], by manual extraction of astaxanthin in dichloromethane/hexane (3:1 *v/v*) and dissolved in dimethyl sulfoxide (DMSO) and spectrophotometric analysis at 530 nm, while Wang et al. (2018) quantified astaxanthin by the same method at a different wavelength (474 nm) [32].

The aim of this work was to test a more accurate and easy method for the quantification of astaxanthin and carotenoids in *H. pluvialis* and also complete a comparison between the spectrophotometric and uHPLC-DAD analysis.

Another important aspect for the improvement of astaxanthin in the food sector is the study of the effect of temperature on its stability. Astaxanthin has been recognized as a thermo-labile molecule and its stability could be affected under a cooking process [33,34] when astaxanthin was absorbed in the flesh of salmonids. Temperature also played a functional role in the extraction of astaxanthin from *H. pluvialis* performed using GRAS (generally recognized as safe) organic solvents such as ethanol and acetone [35,36] or by supercritical fluids using CO_2_ [37,38]. In these operating extraction conditions, temperature was considered in mild conditions or at temperatures between 40 and 100 °C which could also affect the stability and the antioxidants properties of the extracted astaxanthin.

The first studies on the effect of temperature on astaxanthin were carried out on a synthetic form, testing how the all-trans isomer was degraded at temperatures of 25 to 35 to 50 °C for 30 h in the presence of solvents such as dimethyl sulfoxide, dichloromethane, chloroform, acetone, methanol, acetonitrile, and a mixture of dichloromethane and methanol (25:75) [39], or at higher temperatures in the range of 10–70 °C for longer periods such as 60 h [40]. The degradation process was already studied by testing only the all-trans-astaxanthin isomer and its concentration trend at different temperatures and over time. The effect of temperature on natural forms of astaxanthin was tested on an astaxanthin-rich oleroesin produced by the yeast *Phaffia rhodozyma* dispersed in an aqueous solution of propylene glycol at temperatures of 40, 50, 60, 70, or 80 °C for 2 h [41]. The authors pointed out that the degradation effect was more evident within 1.5 h and that it tended to slow down from this time until the end of the experiment (5 h), eventually obtaining a degradation of about 50% of the astaxanthin content at the highest temperatures such as 70 and 80 °C.

The effect of the temperature on the stability of the astaxanthin contained in *H. pluvialis* was also evaluated at milder temperatures such as 21 °C by Raposo et al. (2012) [42], and 4, 20, and 37 °C by Ahmed et al. (2015) [43], for 10 and 21 weeks, respectively, on a biomass after a drying treatment. *H. pluvialis* previously underwent a process of spray-drying inlet/outlet temperatures of 180 and 110 °C, and then temperature storage was tested on a powder spray-drying biomass. Studies on the effect of temperature were also carried out on shrimp extracted astaxanthin (*Litopenaeus vannamei*) [44] and their by-products [45] at temperatures such as 5, 25, and 45 °C for a period of eight weeks or 120 days.

In this work, unlike the others already reported in the literature, the effect of temperature was tested on the stability of a natural form of astaxanthin, produced by *H. pluvialis* with a purity ≥98%, which according to our knowledge has not yet been investigated in a temperature range between 40 and 80 °C and for a time longer than 16 h. The effect of the temperature was also tested on the same sample stored at room temperature for seven days. In addition, the effect of the temperature was assessed on the total astaxanthin content and individual isomers.

## 2. Materials and Methods

Astaxanthin analytical grade was purchased from Sigma-Aldrich (SML0982 SIGMA). This analytical standard is a natural form of astaxanthin obtained from the microalgae *Haematococcus pluvialis* with a purity ≥98%. *H. pluvialis* red phase lyophilized biomass was purchased from the company MICOPERI BLUE GROWTH^®^ (Ravenna, Italy) and β-carotene (22040 Sigma) and lutein (07168 Sigma-Aldrich) analytical standards were purchased from Sigma-Aldrich. Butylated hydroxytoluene (BHT) antioxidant and solvent as dimethyl sulfoxide (DMSO), dichloromethane, hexane, acetonitrile, methanol, and H_2_O uHPLC grade were also purchased from Sigma-Aldrich. All standards and biomass were stored in the dark at −20 °C until use.

### 2.1. Experimental Design

**Figure 2:**
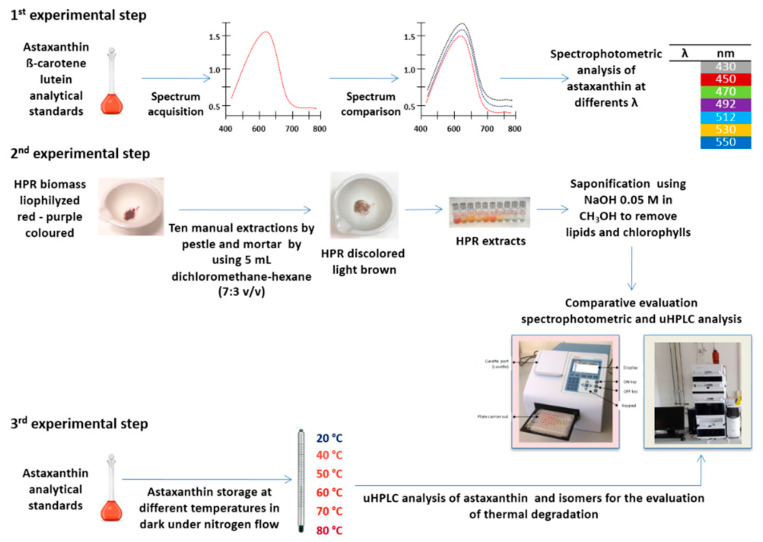
Experimental design.

Experimentation was planned and divided into three different steps as illustrated in Figure 2. The first step concerned the evaluation of the accuracy of the spectrophotometric analysis of astaxanthin, the second step was a comparative evaluation between spectrophotometric and liquid chromatographic analysis by means of uHPLC for the quantification of carotenoids in the microalgae *H. pluvialis*, and the third step was related to the evaluation of the effect of temperature on the concentration of astaxanthin and its isomers.

For the first experimental step, astaxanthin, lutein, and β-carotene analytical standards were dissolved in DMSO to prepare standard solutions at concentrations of 0.5, 5, and 10 mg/L, which were kept before the analysis at room temperature (~20 °C) in order to avoid its crystallization. The spectrophometric analyses were carried out measuring the absorbance in the range 400–800 nm using a Thermo Fisher Scientific Multiskan^®^ produced by Thermo Fisher Scientific Inc. for the evaluation of astaxanthin, lutein, and β-carotene. The instrument was controlled as a standalone with straightforward internal software for quick and simple measurements, or with SkanIt Software for Microplate Readers for PC control which controlled all the instrument functions and provided data processing, as well as reporting functions. The front view of the Multiskan GO with cuvette instrument is shown in Figure 2. Each spectrum was compared to identify the wavelengths for the maximum absorbance of astaxanthin, lutein, and β-carotene. Individual readings of astaxanthin concentrations were also carried out at different wavelengths, including the wavelength for the maximum absorbance, as reported in Figure 2, to evaluate the more suitable and accurate as reported by Li et al. (2012) [31].

The second experimental step was carried out to estimate the total carotenoids in the microalgae *H. pluvialis* red phase (HPR) comparing spetrophotometer and uHPLC analysis. The total carotenoids were extracted as reported by Li et al. (2012) [31]. A known quantity of lyophilized HPR biomass (~20 mg) was ground using a mortar and pestle for ten times, until the biomass lost its red-purple color, as shown in Figure 2. The manual extraction was carried out using 5 mL of a mixture of dichloromethane/hexane at a 7:3 volumetric ratio for 5 min before centrifuging the pellets extract at 400 rpm for 5 min. Then, the supernatant was separated from the pellets and the manual extraction process was repeated. After manual extraction, all the extracts were mixed and only an aliquot of 5 mL was diluted using 95 mL of DMSO, in line with the procedure described by Li et al. (2012) [31]. The samples were saponified using NaOH 0.05 M in CH3OH to remove lipids and chlorophylls from the sample, then, after about 6 h, neutralized with NH4Cl 0.5 M and analyzed with spectrophotometer and uHPLC Agilent 1290 Infinity II.

### 2.2. uHPLC Analysis

Chromatographic analysis of carotenoids was performed using an Agilent 1290 Infinity II uHPLC coupled with a diode array detector (DAD). Carotenoids were detected in the range 400–700 nm and at 444, 450, and 478 nm, as reported by Di Sanzo et al. (2018) [37] and separated in uHPLC by means of a C18 column (Agilent Zorbax Eclipse plus C18 colum, 50 mm length, internal diameter 2.1 mm, 1.8 μm particle size, part number 959741-902). The following chromatographic conditions were set for the identification of carotenoids: an isocratic phase methanol/water (95:5 *v/v*), a flow rate of 0.4 mL/min, and a temperature of 28 °C [46]. Astaxanthin, β-carotene, and lutein analytical standards were dissolved in chloroform with 0.1% BHT as antioxidant.

### 2.3. Thermal Degradation of Astaxanthin

The effect of temperature on the stability of astaxanthin was tested using an analytical standard (SML0982 SIGMA, purity ≥98%). The astaxanthin standard was dissolved in chloroform and stored in the absence of light and air in an amber glass tube, which was placed in a thermostatically controlled water bath to carry out thermal degradation tests. The experimental test was carried out to investigate the thermal degradation at different temperatures between 40 and 80 °C and time as reported in Table 1. For each test, the sample was stored for a maximum of 16 h. The total astaxanthin content and all-trans, 9-cis, and 13-cis isomers were quantified by uHPLC at fixed time intervals of 1, 8.5, and 16 h.

The thermal degradation effect was also tested at room temperature, astaxanthin analytical standard was located at a temperature of 20 °C, in the dark in the absence of air in a water bath controlled for a longer period of up to 7 days. The total astaxanthin concentration was analyzed at fixed interval time at 0.5, 2, 5, and 7 days.

### 2.4. Statistical Analysis

Each test was performed in triplicate. Data elaboration (mean and standard deviation) and statistical analysis (one-way ANOVA) were carried out by means of SigmaStat 4.0 statistical software (Systat Software Inc., San Jose, CA, USA). The level of significance was taken as *p* ≤ 0.05, Dunnett’s and Tukey test were performed on rank, the first compared results with a control treatment, and the second compared results with the treatments.

## 3. Results

### 3.1. Astaxanthin and Carotenoids Characterization

**Figure 3:**
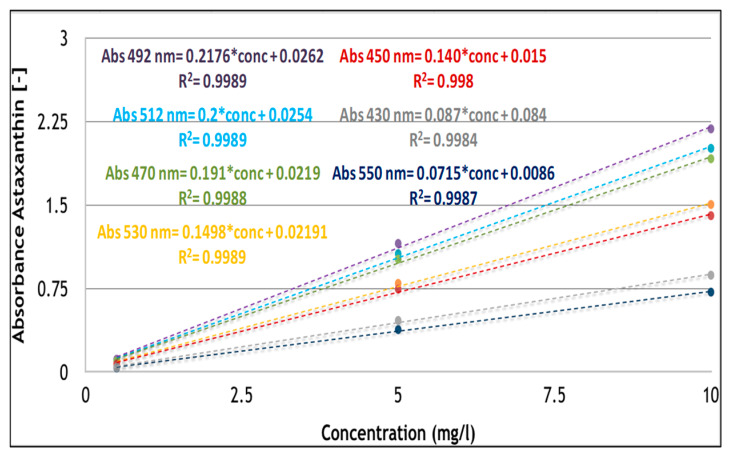
Calibration curves of astaxanthin in dimethyl sulfoxide (DMSO) (0.5, 5, and 10 mg/L for λ1 = 430, λ2 = 450, λ3 = 470, λ4 = 492, and λ5 = 510, λ6 = 530, and λ7 = 550 nm).

The most appropriate wavelength for the quantification of astaxanthin by spectrophotometer was evaluated using a standard astaxanthin derived from *H. pluvialis* (purity ≥ 98%). Astaxanthin was dissolved in DMSO at three different concentrations and the absorbance was measured at different wavelengths (λ) in the range of 430–550 nm, in accordance with the characteristics of the astaxanthin absorption spectrum. The obtained calibration curves, as observed in Figure 3, showed a correlation coefficient R^2^ higher than 0.998 in all cases.

The wavelength of maximum absorbance (λ_max_) was confirmed at 492 nm, as observed by different authors for astaxanthin dissolved in DMSO [31,47]. Astaxanthin maximum absorbance can be also detected at different values such as 480, 478, and 488 nm, when extraction solvents such as acetone, ethanol, and hexane, respectively, have been used [1]. The linear regression equations obtained for astaxanthin in DMSO were compared with those obtained by other authors in the same condition, as reported in Table 2 [31]. A full correspondence is showed confirming the properties of astaxanthin dissolved in DMSO at the fixed wavelengths as proposed in the literature (Table 2).

Furthermore, astaxanthin absorbance was also analyzed at wavelengths below 492 nm, such as 430, 450, and 470 nm which were not already evaluated [31]. These wavelengths, that are most appropriate for carotenoids such as β-carotene and lutein were selected to investigate the response of astaxanthin and possible overestimation or underestimation of astaxanthin quantification by spectrophotometer.

**Figure 4:**
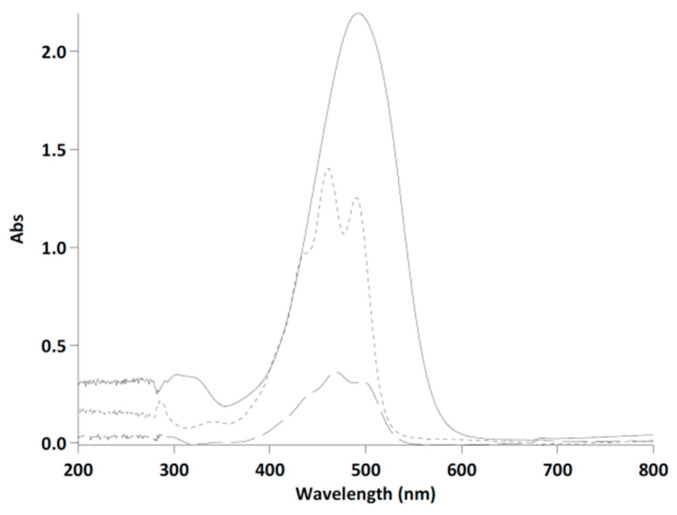
Absorbance spectra of astaxanthin (continuous line), lutein (narrow dotted line), and β-carotene (wide dotted line) at a concentration of 10 mg/L in DMSO.

The spectrum acquisition of β-carotene, lutein, and astaxanthin standard (10 mg/L concentration) dissolved in DMSO were obtained in the range 200–800 nm, as shown in Figure 4. The maximum absorbance of lutein was around 460 nm and for β-carotene equal to 466 nm. The different absorbance intensity registered for the three standards was due to the different solubility in DMSO equal to 1000 mg/L for lutein and 30 mg/L for β-carotene [48]. Although astaxanthin, lutein, and beta-carotene had a maximum absorbance at different wavelengths, as shown in Figure 4, at the value of maximum absorbance of astaxanthin (492 nm), lutein and β-carotene can also absorb interfering with astaxanthin.

It was observed by Li et al. (2012) that other species such as siphonaxanthin and violaxanthin did not absorb at wavelengths exceeding 530 nm [31]. For this reason, it is evident that the use of the calibration curve at the maximum wavelength of the astaxanthin (492 nm) was not appropriate for the quantification and risked running into an overestimate. However, as can be seen from Figure 4, astaxanthin in the range 530–550 nm still had quite significant absorbance values, whereas, in this range, lutein and β-carotene absorbance was very slight. The previous consideration shown as the wavelength of 530 nm was considered to be more appropriate for the spectrophotometric analysis of astaxanthin as compared with 492 nm, which was also evidenced by Li et al. (2012) [31].

After the evaluation of the most appropriate wavelength for the spectrophotometric analysis of astaxanthin, carotenoids were manually extracted from the microalgae *H. pluvialis* red phase and quantified by means of a spectrophotometer and uHPLC-DAD. The astaxanthin content was expressed as mg/L and mg/g, as reported in Table 3, distinguishing the results from spectrophotometric and uHPLC-DAD analysis.

These values were comparable with respect to astaxanthin concentration and quantity analyzed by the spectrophotometer and uHPLC with a content of approximately 20.00 ± 0.05 mg/g of astaxanthin in *H. pluvialis* red phase that corresponded to approximately 2% dry weight.

**Figure 5:**
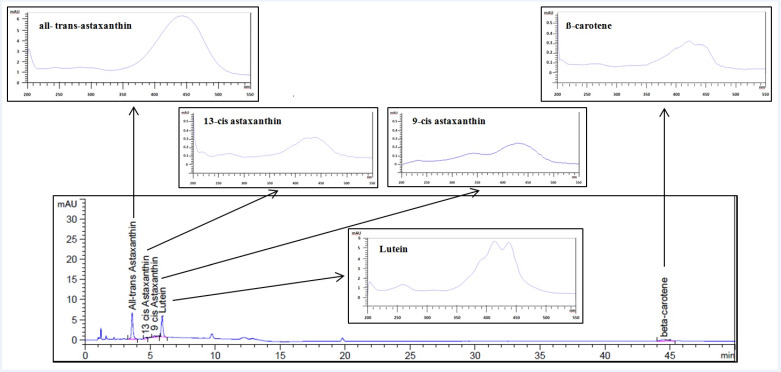
Chromatogram of the *H. pluvialis* sample analyzed and related absorption spectra of all-trans-astaxanthin, 9-cis astaxanthin, 13-cis astaxanthin, lutein, and β-carotene. Operative condition: Zorbax reverse phase C18 column with 0.4 mL/min of methanol/water (95:5, *v/v*) as a mobile phase.

In addition to the amount of astaxanthin, the content of carotenoids was analyzed directly using uHPLC-DAD. The chromatogram and the relative absorption spectra of each of the carotenoids with their characteristic shape are shown in the Figure 5.

The uHPLC-DAD analysis was used to assess the prevailing species contained in the *H. pluvialis* red phase, astaxanthin, lutein, and β-carotene and also the separation of all-trans, as well as 9-cis and 13-cis isomers of astaxanthin (Figure 5). The obtained results of the quantification are reported in Table 4. With regard to the astaxanthin content in *H. pluvialis*, all-trans-astaxanthin isomer resulted as the most abundant with respect to cis-isomers with a content equal to 8.30 ± 0.04 mg/g followed by 9-cis and 13-cis astaxanthins with a value equal to 6.24 ± 0.05 mg/g and 5.46 ± 0.06 mg/g, respectively. Lutein accounted for 7.70 ± 0.04 mg/g and β-carotene for 0.98 ± 0.04 mg/g. Adding the single carotenoids quantified in uHPLC-DAD, it was possible to obtain the content of the total carotenoids present in *H. pluvialis* equal to 28.70 ± 0.05 mg/g dry weight corresponding to a percentage equal to 2.87% ± 0.15%.

### 3.2. Thermal Stability of Astaxanthin

**Figure 6:**
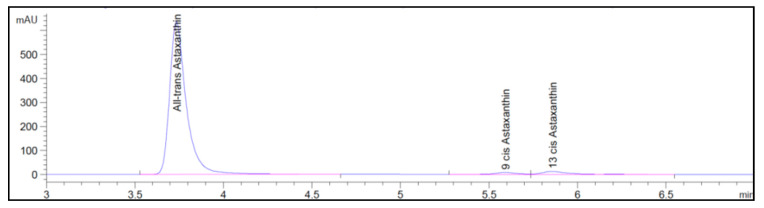
uHPLC-DAD chromatogram of initial astaxanthin sample. All-trans-astaxanthin, 9-cis, and 13-cis astaxanthin isomers peaks were identified.

Thermal stability of astaxanthin was tested in a temperature range of 40 to 80 °C and over time at specific intervals of 1, 8.5, and 16 h. The initial concentration (C_0_) of astaxanthin had the characteristics as shown in Figure 6.

Three isomers were identified in uHPLC-DAD. The all-trans-astaxanthin isomer had an average isomeric ratio of 96.2% ± 0.75%, followed by 9-cis astaxanthin and 13-cis astaxanthin with an average isomeric ratio equal to 1.23% ± 0.15% and to 2.30% ± 0.235, respectively.

The all-trans-astaxanthin isomer concentration was 59.42 ± 0.34 mg/L and 9-cis and 13-cis isomers were 0.85 ± 0.10 mg/L and 1.58 ± 0.15 mg/L, respectively, whereas the total concentration of astaxanthin was equal to 61.85 ± 0.79 mg/L (Table 5). These concentrations were considered to be a kind of “control” with respect to the results obtained in the experimental tests.

**Figure 7:**
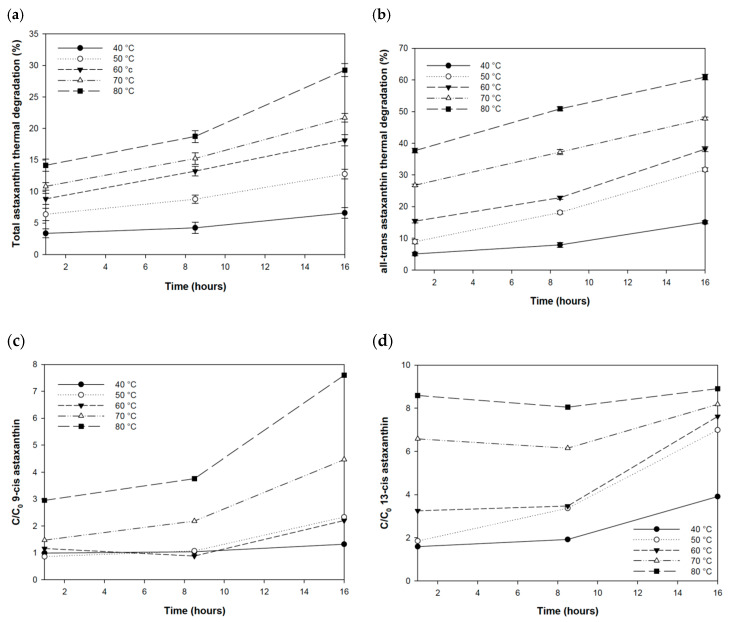
Thermal degradation effect of astaxanthin expressed as percentage (±SD, *n* = 3) for total content (**a**); all-trans isomer (**b**); and the ratio of the final (C) to the initial (C_0_) concentration of the 9-cis astaxanthin (**c**); and 13-cis astaxanthin (**d**) isomers.

Astaxanthin and isomer concentrations were subsequently quantified on the samples stored in a thermostatically controlled water bath for each tested temperature in the range 40–80 °C. The effect of temperature on astaxanthin was expressed as percentage degradation (Figure 7a). In general, the effect of temperature was predominantly observed at higher temperatures, i.e., above 50 °C. After one hour of storage, the highest percentage of degradation was observed at 70 and 80 °C when astaxanthin concentration decreased 10.8% ± 0.59% and 14.2% ± 0.97%, respectively. A significant decrease was also observed at the other tested temperatures (*p* < 0.001), as highlighted by the analysis of variance (one-way ANOVA) performing Dunnett’s test. For all temperatures, the thermal degradation increased over time, reaching the maximum effect at 80 °C after 16 h with a decrease of 29.3% ± 1.02%, whereas the minimum effect was reached at 40 °C equal to 6.6% ± 0.86%.

The temperature affected the concentration of the total astaxanthin, and also the concentration of the trans and cis isomeric forms. At all temperatures between 40 and 80 °C, over time, it was possible to observe a decrease in the concentration of the all-trans isomer (Figure 7b) and an increase in the concentration of the 9-cis and 13-cis astaxanthin isomers (Figure 7c–d). This trend was typical of an isomerization process where the all-trans-astaxanthin isomer was converted into cis forms, which occurred due to the effect of temperature and other factors such as light and air exposure. According to the authors Yuan and Chen (1999) [39], the astaxanthin isomerization process was reversible since there was no total conversion of the all-trans-astaxanthin isomer into cis forms and it did not follow a constant trend over time. It occurs faster in the early storage periods and slows down when a balanced situation is reached. All-trans-astaxanthin isomer underwent the effect of thermal degradation even after an hour of storage at 80 and 70 °C showing a degradation equal to around 37.7% ± 0.78% and 26.76% ± 0.38%, respectively (Figure 7b). Although the effect of degradation was very evident at higher temperatures, a statistically significant difference was observed for all temperatures (*p* < 0.001 one-way Anova Dunnett’s test). Contrary to the decrease of all-trans-astaxanthin isomer, the concentration of the 9-cis astaxanthin and 13-cis astaxanthin isomers increased, respectively, by 3.5 times and 5.4 times as compared with the initial concentration (Figure 7c–d).

As observed for the total astaxanthin, the effect of thermal degradation on the all-trans-astaxanthin isomer increased concomitantly with time from 1 to 16 h of storage, with a significant trend for all fixed temperatures (*p* < 0.001 one-way Anova Tukey test). The higher degradation of all-trans-astaxanthin to 60.9% ± 0.89% was reached after 16 h at 80 °C. This condition corresponded to an increase of nine times of 9-cis isomer. The 9-cis isomer appeared to be the most influenced by the isomerization process while the 13-cis isomer increased in concentration over time at all tested temperatures except at 80 °C (*p* > 0.05 one-way Anova Tukey test).

**Figure 8:**
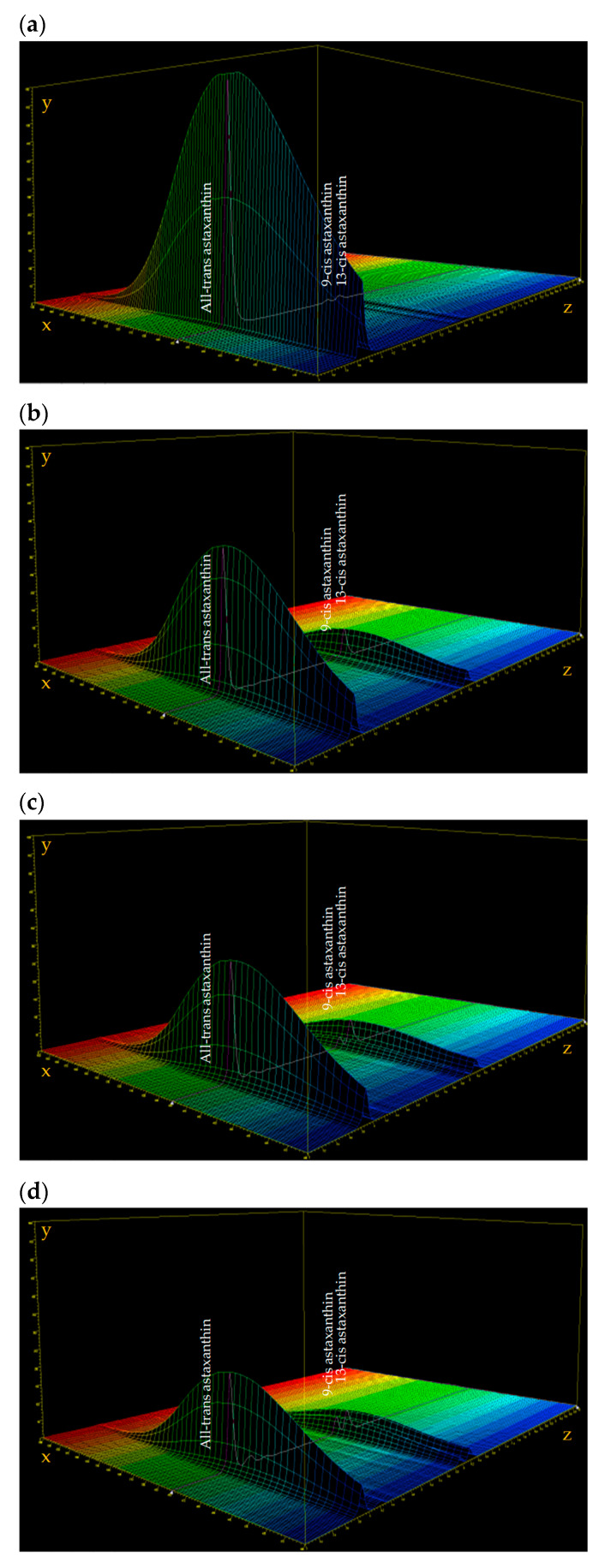
uHPLC-DAD chromatogram and spectra three-dimensional (3D) plot of initial astaxanthin concentration (**a**); astaxanthin stored after 16 h at 60 °C (**b**); at 70 °C (**c**); at 80 °C (**d**). The x-axis described the spectrum in the range of 400–600 nm, the y-axis described the UV intensity in the range 0–600 mAU, and the z-axis described the time (0–10 min).

The isomerization process is even more evident observing the three-dimensional (3D) plots of the chromatograms and spectra of the initial astaxanthin concentration (C0), and astaxanthin at 60, 70, and 80 °C after 16 h. In Figure 8a, the 3D plot of the initial concentration (C0) is shown, in which the initial peak of the all-trans isomer and the subsequent peaks of the 9-cis and 13-cis isomers are visible along the z-axis. Along the x-axis, the absorption spectrum of each isomer is shown, with a maximum absorbance of 478 nm. As can be seen in Figure 8b–d, the peak height of the all-trans isomer decreased significantly in samples incubated at 60, 70, and 80 °C after 16 h of storage, with an increase in the peak height of the 9-cis and 13-cis isomers.

Thermal degradation of astaxanthin was also investigated at room temperature and for a longer period of up to seven days. As reported in Table 6, thermal degradation of astaxanthin was observed also at room temperature with a slight linear trend.

The concentration of astaxanthin decreased over time reaching a decrease of 7.07% ± 0.44% decrease after seven days of storage.

## 4. Discussion

In the literature, astaxanthin derived from *H. pluvialis* has been mainly quantified spectrophotometrically at different wavelengths such as 472 nm [25], 474 nm [26], and 480 nm [27] after an extraction by using acetone as the solvent. In addition, other authors tested the extraction of astaxanthin with two types of solvent, a mixture of methanol/dichloromethane (3:1, *v/v*) and DMSO and using a spectrophotometer at 474 nm [32]. The wavelengths that are used for the spectrophotometer analysis of astaxanthin usually correspond to the maximum absorbance of astaxanthin. However, as shown in this study and by other authors [31], the use of maximum absorbance would not be as appropriate as the absorption spectrum of other carotenoids overlapped.

In order to avoid this overlap problem, the proposed analytical method by manual extraction of total carotenoids from *H. pluvialis* by methanol/dichloromethane (3:1, *v/v*) and uHPLC-DAD analysis highlighted the presence of astaxanthin and other carotenoids. The more detailed analysis of carotenoids’ composition in microalgae, such as *H. pluvialis,* is certainly an interesting aspect given the antioxidant properties of these compounds. Astaxanthin and its isomers corresponded to 69.69% of total carotenoids contained in *H. pluvialis,* whereas lutein represented about 27% and only a small percentage, equal to about 3%, was relative to the β-carotene, as also reported by other authors [30]. The contribution of astaxanthin as compared with other carotenoids also reached a percentage of about 81% of total carotenoids, whereas lutein and beta-carotene also comprised percentages lower than 0.5% and 1% [11]. In addition, the amount of total carotenoid quantified by the proposed method resulted in accordance with literature data [28,29,30,49]. Some authors, in fact, quantified the red cysts of *H. pluvialis* between 1.79% and 2.00% of total carotenoids as compared with dry weight [29] or 1.9%–2% dry weight [30]. In addition, total carotenoids were quantified between 10 and 40 mg/g dry weight [28,49] or up to a maximum of almost 6.8 mg/L [27].

By comparing the results obtained in this work and those reported in the literature, the proposed method for total carotenoids identification and quantification emerges as a valid methodology. The application of liquid chromatography in the proposed methodology allowed the analysis of total carotenoids content and composition. Every single carotenoid and their total quantification can be detected more precisely than the spectrophotometric methods currently used such as the methods by Davies (1976) [23] and Lichtenthaler (1987) [24]. Furthermore, the identification of astaxanthin and its isomers (all-trans, 9-cis, and 13-cis) that can occur through the proposed methodology is fundamental for their antioxidant properties that have been investigated by several authors [50]. Yiang et al. (2019) highlighted the anti-inflammatory effects of astaxanthin isomers for both all-trans and cis [50].

Thermal degradation of astaxanthin produced by *H. pluvialis* was exploited principally at temperatures above 37 °C for its applications in the food sector. The effect of high temperatures under different cooking conditions, such as 600 W microwave, and boiling and frying at 160 °C for different exposure times (3, 10, 15, and 20 min) showed that the total content of astaxanthin decreased by 70% after 20 min for all types of cooking except for frying in which it was degraded by 100% [51].

The temperature effect was also tested on the stability of astaxanthin oleoresin produced by the *Phaffia rhodozyma* yeast [41] at different temperatures between 40 and 80 °C decreased by 50% at temperature above 60 °C with a slower degradation process until the end of the experiment (5 h). Thermal stability of astaxanthin produced by *H. pluvialis* was tested for a long time and was more stable, observing a maximum decrease of 30% at a temperature of 80 °C after 16 h of incubation. Furthermore, the trend of thermal degradation increased over time without any slowdown in the degradation process, as observed by Villalobos-Castillejos et al. (2013) [41].

The effect of temperature on all-trans-astaxanthin isomer and the isomerization process was investigated by different authors [40,45]. In this work, the decreasing trends observed at 70 °C after 8.5 h and at 60 °C after 16 h of all-trans-astaxanthin isomer are comparable with respect to the literature data [40]. In addition, a process of isomerization was observed refluxing astaxanthin for 0, 1.5, 3, and 15.5 h [52]. The isomeric percentages at 70 °C after 16 h were 59% for all-trans isomers, 7% and 24% for 9-cis and 13-cis isomers, respectively, in line with other observed isomerization processes at the same temperature and time [52]. The effect of temperature on the degradation of astaxanthin is also an important aspect with regard to the extraction processes by means of organic solvents. In this context, the maximum extraction yield of astaxanthin extracted from *H. pluvialis* in acetone and ethanol by pressurized liquid extraction (PLE) was obtained at 40 °C and 67 °C, although higher temperatures, up to 100 °C, were tested [36]. The investigation of thermal degradation of astaxanthin contributes to the explanation of the interference of temperature with respect to extraction yield in astaxanthin extraction processes.

The astaxanthin stability was also affected at low temperatures such as 20 °C. Freeze-dried *H. pluvialis* biomass incubated under normal conditions at 20 °C decreased 35% after three weeks and 81% after 20 weeks [43]. A very low degradation rate equal to 1% on the content of synthetic and natural astaxanthin produced by the shrimp by-products at 25 °C for eight weeks was also observed in an additional work [44]. A discrepancy was observed with respect to results obtained and literature data, thus, thermal degradation of astaxanthin with a maximum of 7% after one week, demonstrated the need to test the thermal degradation of astaxanthin at room temperature for longer time intervals.

## 5. Conclusions

Natural rich-carotenoids products derived from *H. pluvialis*, in which astaxanthin is the main but not the only compound, need an analytical method to separate and quantify carotenoids. The spectrophotometric method is not fully applicable due to the overlapping problems of carotenoids spectra. The proposed methodology avoids this problem and allows a characterization of carotenoids in *H. pluvialis*. Astaxanthin, which is an antioxidant molecule of thermo-labile nature, is strongly influenced by the effect of temperature, which caused a degradation in terms of a decrease in the concentration of all-trans isomers and an increase in the 9-cis and 13-cis isomers both at room temperature and at higher temperatures lower than those of the cooking process. It is, therefore, necessary to preserve the stability of astaxanthin and the antioxidant role under cooking phenomena and also during storage at room temperature, and under industrial extraction processes.

## Figures and Tables

**Table 1:** Temperature and time of thermal degradation tests.

Test n°	1	2	3	4	5	6	7	8	9	10	11	12	13	14	15
Temperature (°C)	40	40	40	50	50	50	60	60	60	70	70	70	80	80	80
Time (hours)	1	8.5	16	1	8.5	16	1	8.5	16	1	8.5	16	1	8.5	16

**Table 2:** Comparison of linear regression equations of astaxanthin dissolved in DMSO.

Wavelength λ (nm)	Equations in this Work	Literature [31]
492	y = 0.2176x + 0.0262 (1)	y = 0.2220x + 0.0104
512	y = 0.2x + 0.0254 (2)	y = 0.2096x + 0.0110
530	y = 0.1498x + 0.0191 (3)	y = 0.1556x + 0.0107
550	y = 0.0715x + 0.0086 (4)	y = 0.0759x + 0.005

**Table 3:** Comparison of results obtained by spectrophotometer and liquid chromatography for the carotenoids characterization of *Haematococcus pluvialis* red phase (±SD, *n* = 3).

Analysis	Astaxanthin Concentration (mg/L)	Astaxanthin Content (mg/g)
Spectrophotometer	8.34 ± 0.15	20.02 ± 0.20
uHPLC-DAD	8.42 ± 0.04	20.00 ± 0.05

**Table 4:** Concentration and content of principal carotenoids contained in *H. pluvialis* red phase expressed, respectively, as mg/L and mg/g (±SD, *n* = 3).

Carotenoids	Concentration (mg/l)	Content (mg/g)
All-trans-astaxanthin	3.46 ± 0.03	8.30 ± 0.04
9-Cis astaxanthin	2.59 ± 0.05	6.24 ± 0.05
13-Cis astaxanthin	2.27 ± 0.05	5.46 ± 0.06
Total astaxanthin	8.42 ± 0.04	20.00 ± 0.05
Lutein	3.20 ± 0.04	7.70 ± 0.04
β-Carotene	0.41 ± 0.02	0.98 ± 0.04
Total	12.03 ± 0.04	28.70 ± 0.05

**Table 5:** Total astaxanthin and isomers initial concentration (C_0_) in the sample, expressed as mg/L (±SD, *n* = 3).

Astaxanthin Isomers	Concentration (C_0_)
	(mg/L)
All-trans	59.42 ± 0.34
9-Cis	0.85 ± 0.10
13-Cis	1.58 ± 0.15
Total	61.85 ± 0.79

**Table 6:** Total astaxanthin thermal degradation percentage at room temperature (20 °C) for 7 days (±SD, *n* = 3).

Time (days)	Astaxanthin Thermal Degradation
	%
0.6	0.16 ± 0.08
2	2.29 ± 0.13
5	4.74 ± 0.23
7	7.07 ± 0.44
